# Maternal cosmetics use during pregnancy and risks of adverse outcomes: a prospective cohort study

**DOI:** 10.1038/s41598-019-44546-z

**Published:** 2019-05-29

**Authors:** Huixia Li, Jianfei Zheng, Hua Wang, Guangwen Huang, Qun Huang, Na Feng, Juan Xiao

**Affiliations:** 1 Department of Child Health Care, Hunan Provincial Maternal and Child Health Care Hospital, Changsha, 410008 Hunan Province China; 2 NHC Key Laboratory of Birth Defects Research, Prevention and Treatment, Hunan Provincial Maternal and Child Health Care Hospital, Changsha, 410008 Hunan Province China; 3 Department of Emergency and Intensive Care Medicine, Second Xiangya Hospital, Central South University, Changsha, 410011 Hunan Province China; 4 Department of Maternal Health Care, Hunan Provincial Maternal and Child Health Care Hospital, Changsha, 410008 Hunan Province China; 5 Department of Health Care, Shenzhen Nanshan Maternal and Child Health Care Hospital, Shenzhen, 518067 Guangdong Province China

**Keywords:** Epidemiology, Epidemiology

## Abstract

To probe into the associations between maternal personal cosmetics use during pregnancy and risk of adverse outcomes, and explore the potential dose-response relationships, we carried out a prospective cohort study involving 9710 pregnant women in Zhuzhou City and Xiangtan City in Hunan province during 2016–2017. A structured questionnaire was used to collection information for the pregnant women and their pregnancy outcomes. Odds ratios (OR) and 95% confidence intervals (CI) were calculated by binary or multinomial logistic regressions. The study population included 4652 (47.9%) cosmetics non-users and 5058 (52.1%) cosmetics users. Cosmetics use was associated with an increased risk of small for gestational age (SGA) (aOR = 1.23, 95%CI 1.04 to 1.44), compared with cosmetics non-users. A positive dose-response relationship between frequency of cosmetics use and SGA was observed, although a borderline association was found at low use frequency (1–2 times per week; aOR = 1.18, 95%CI 0.99 to 1.40) and moderate use frequency (3–4 times per week; aOR = 1.23, 95%CI 0.92 to 1.64). High-frequency of cosmetics use (≥5 times per week) was significantly correlated with a higher risk of SGA (aOR = 1.83, 95%CI 1.25 to 2.69). No significant association between personal cosmetics use and the risk of preterm birth, low birth weight, macrosomia, or large for gestational age was observed. The present study suggests that personal cosmetics use will increase the risk of SGA, but further research is required to determine which cosmetic products may account for the higher risk of SGA.

## Introduction

Cosmetic products are necessities in our daily life and mostly used by young women, including pregnant women. Recent cross-sectional studies show 60–80% of pregnant women also use cosmetic products, including facial cleaner, day cream, foundation, mascara, lipstick, eye pencil, eye shadow and make-up remover^1–3^. Cosmetics products contain various chemical substances (e.g. phthalates, parabens, formaldehyde, dioxane, nitrosamine, hydroquinone, phenol, organic solvents) and heavy metals (e.g. Pb, Hg, As, Cd, Cr, Sb, Ni), which are either added purposely as efficacy components to enhance the skin care effect of cosmetic products or brought in from the raw materials, auxiliary materials or technical problems during the production process^4–7^. Cosmetics use is reportedly related with increased personal exposure to parabens, phthalates and benzophenone^2–4,8,9^.

Since the majority of cosmetic products are used directly onto skins, the hazardous chemicals can directly pass the epidermal barrier to the dermis of the human body and thereby enter the systemic blood circulation, which potentially damage the fertility and reproductive health of women. Some of these chemicals are related to adverse pregnancy outcomes. For example, a French case-control study shows maternal prenatal exposure to phenols (2,4-dichlorophenol and 2,5-dichlorophenol) is inversely correlated with male birth weight^10^, and two studies suggest pregnant exposure to phthalates is associated with preterm birth or pregnancy loss^11,12^.

Previous studies show occupational exposure of pregnant women to cosmetic products (e.g., hairdressers and cosmetologists) is associated with miscarriage, perinatal mortality, preterm birth, low birth weight, and small for gestational age (SGA) and other adverse pregnancy outcomes^13–17^. A retrospective cohort study in New York State implies that licensed cosmetologists are prone to have low birth weight babies compared to licensed realtors^15^. Two registry-based studies indicate an elevated risk of SGA for cosmetologists compared to other working women^14,16^. A recent meta-analysis confirms cosmetologists are under higher risk of infertility, fetal death, and preterm birth than the general population^17^. However, these studies focus on adverse pregnancy outcomes of occupational exposure to cosmetic products, but not personal exposure.

So far as we know, there is no study reporting the relationship between maternal cosmetics use (personal use of cosmetics) during pregnancy and risks of adverse pregnancy outcomes. This large Chinese prospective cohort study was aimed to examine such associations and potential dose-response relationships. The adverse pregnancy outcomes concerned included preterm birth, low birth weight, macrosomia, SGA and large for gestational age (LGA).

## Methods

### Study population

Pregnant women at 16 ± 2 weeks of gestation were recruited from local midwifery hospitals (e.g. community hospitals, general hospitals, traditional Chinese medicine hospitals, perinatal care centers) in Zhuzhou City and Xiangtan City, Hunan Province, China, between September 2016 and August 2017. The follow-up period lasted from the first antenatal care visit to 42 days after delivery.

Inclusion criteria were: (1) singleton pregnancy; (2) residence in the study areas during the follow-up period; (3) explicit cosmetics use status and frequency; (4) provision of informed consent. Exclusion criteria were: (1) miscarriage or termination of pregnancy; (2) infants with congenital anomalies; (3) infants with missing gestational age, birth weight or sex; (4) gestational age older than 42 weeks; (5) profession as hairdresser or cosmetologist.

### Data collection

During prospective follow-up, the data concerning the pregnancy outcomes of pregnant women and the birth of infants were acquired by research midwives who had underwent a unified training. The status of cosmetics exposure was determined by directly asking each pregnant woman on the recruitment day if she used cosmetics during pregnancy. According to the self-reported use or no-use of cosmetic products at any time during pregnancy, the pregnant women were divided into two groups: cosmetics users and cosmetics non-users. The data of pregnancy outcomes were acquired by using a structured questionnaire. At the first antenatal care visit, research midwives interviewed every recruited pregnant woman face-to-face to collect demographic and clinical characteristics, including maternal age, residence, ethnicity, education level, occupation, parity, abnormal reproductive history and pre-pregnancy body mass index (BMI), pregnancy comorbidity and date of last menstrual period (LMP). After delivery, all infants were examined within the first 24 h by neonatologists in these hospitals. Information regarding infants was recorded opportunely, including birth weight, gestational age at birth, sex, and occurrence of congenital anomaly.

### Cosmetics exposure

Cosmetics use was defined as having used any personal face hygiene care products (e.g., facial cleaner, astringent, smoothing toner, moisturizer, day cream, night cream, sun screen, facial scrub and facial mask) or make-up products (e.g., foundation, blush, concealer, shading powder, luminizer, brow pencil, brow powder, eye shadow, eye pencil, mascara, lipstick, lip liner, lip gloss, make-up remover, nail polish, nail saver, nail polish remover and perfume) during pregnancy. Only personal use of cosmetic products was considered, but not occupational exposure. The use of cosmetics was evaluated according to general use habits. Cosmetics users were asked about the use frequency, which was divided into low, moderate and high frequency with 1–2, 3–4, and ≥5 times per week, respectively.

### Definitions of covariates

Maternal age was classified into five scales: ≤20, 21–25, 26–30, 31–35 and >35 years. Residence was divided into urban and nonurban residences. Ethnicity was classified into Han and minorities (the other 55 ethnicities in China except Han). Education level was classified (1) primary school and below, (2) middle school, and (3) college and above. Occupations included farmer, worker, administrative staff, and business/company staff and else. History of abnormal reproduction covered miscarriage, stillbirth, neonatal death, preterm birth, low birth weight or congenital anomaly. Women with pre-pregnancy BMI <18.5, 18.5–25.0, and ≥25.0 kg/m^2^ were classified as underweight, normal weight and overweight, respectively (BMI = weight just before pregnancy divided by height squared)^18^. Pregnancy comorbidity was defined as pregnant women had suffered from acute or chronic disease during pregnancy, such as hypertension, diabetes, heart or liver or kidney disease, hyperthyroidism, hypothyroidism, anemia, etc. These covariates were considered as potential confounders probably influencing the relationship between cosmetics use and adverse pregnancy outcomes and should be controlled in the analysis.

### Outcome measures

Birth weight was defined as the weight of each infant measured within 24 hours of birth. Gestational age was defined as the duration of pregnancy from the first day of LMP to birth. If LMP was uncertain, gestational age was estimated by sonography. Birth weight <2500 g and ≥4000 g were considered as low birth weight and macrosomia respectively^19^, and birth <37 gestational weeks was regarded as preterm birth^15^. Birth weight <10th, 10th–90th, and >90th percentile were defined as SGA, appropriate for gestational age (AGA) and LGA, respectively^18^, adjusted for gestational age and sex using Chinese standards (enacted by Chinese neonatologists as a reference for Chinese population)^20^.

### Statistical analysis

Proportions of maternal characteristics and prevalence rates of adverse pregnancy outcomes were calculated among different groups. Differences of maternal characteristics between cosmetics non-users and users were determined using Chi-square test. Unadjusted and adjusted odds ratios (OR) and 95% confidence intervals (CI) were calculated by binary logistic regression for preterm birth, and by multinomial logistic regression for low birth weight/macrosomia and SGA/LGA (infants with normal birth weight and AGA were the controls for the dependent variables, respectively). In order to control the potential confounders, we used multivariable logistic regression models to adjust maternal age, residence, ethnicity, education level, occupation, parity, abnormal reproductive history, pre-pregnancy BMI and pregnancy comorbidity. Dose-response relationship was tested by exploring the associations between frequency of cosmetics use and adverse pregnancy outcomes. The above results were referred to as relative risk (RR), because OR is a good approximation for the risk ratio in the case of rare outcomes^21^. Statistical analyses were performed on SPSS 19.0 (IBM, Chicago, IL, USA). All statistical tests were two-tailed at the significance level of *P* < 0.05.

### Ethics statement

This study was conducted in accordance with the Declaration of Helsinki with ethical approval from the Ethics Committee of Hunan Provincial Maternal and Child Health Care Hospital. Written informed consents were obtained from all the pregnant women.

## Results

**Figure 1:**
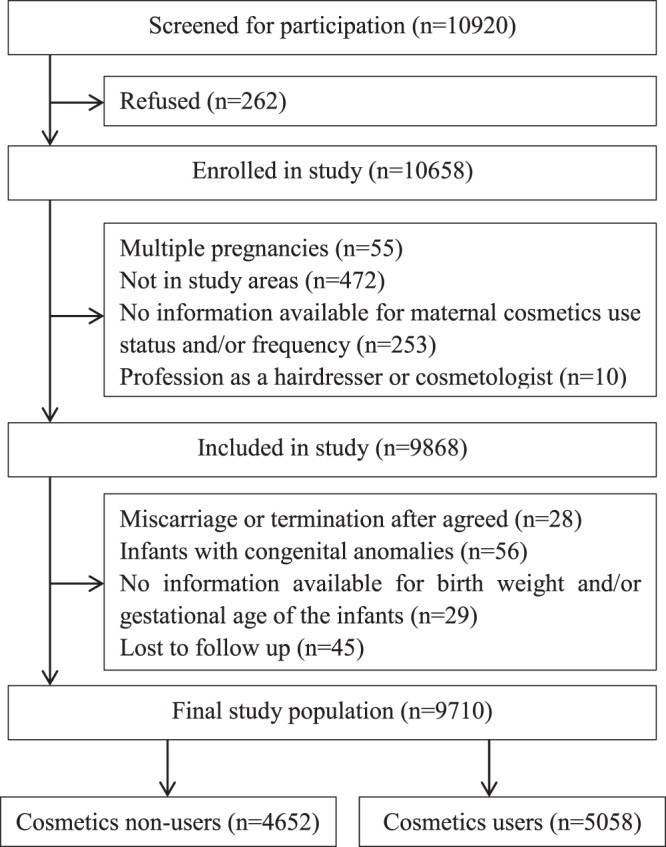
Participants recruited and final study population.

From the initial 10920 pregnant women screened for participation, 262 (2.4%) refused to be enrolled in the study, and 10658 (97.6%) agreed to be enrolled in the study; out of the enrolled pregnant women, 253 (2.3%) had no information available for maternal cosmetics exposure mainly due to an ambiguous frequency of cosmetics use (Fig. 1). A total of 9868 pregnant women met the inclusion criteria at enrollment, but only 9710 were included in the final analysis. During the interviews at 16 weeks of gestation, 4652 (47.9%) pregnant women reported that they did not use any cosmetics during pregnancy, and 5058 (52.1%) used some cosmetics during pregnancy. These figures represent an overall loss to follow up frequency of 0.5% during pregnancy.

### Characteristics of cosmetics non-users and users

Table 1 shows the demographic and clinical characteristics of the two groups. The two groups were significantly different in maternal age, residence, education level, occupation, parity and pre-pregnancy BMI, but not in ethnicity, history of abnormal reproduction or pregnancy comorbidity. Cosmetics non-users were more likely to be farmers, have lower education level and be of higher parity than cosmetics users.

**Table 1:** Characteristics of cosmetics non-users and users.

Characteristics	Cosmetics non-users (N = 4652)	Cosmetics users (N = 5058)	*χ* ^2^	*P* Value
Maternal age			52.853	0.000
≤20 yr	142 (3.1)	148 (2.9)		
21–25 yr	1918 (41.2)	2275 (45.0)		
26–30 yr	1719 (37.0)	1922 (38.0)		
31–35 yr	618 (13.3)	559 (11.1)		
>35 yr	255 (5.5)	154 (3.0)		
Residence			71.031	0.000
Urban	450 (9.7)	777 (15.4)		
Nonurban	4202 (90.3)	4281 (84.6)		
Ethnicity			1.269	0.260
Han	4599 (98.9)	5012 (99.1)		
Minorities	53 (1.1)	46 (0.9)		
Education level			59.028	0.000
Primary school and below	135 (2.9)	71 (1.4)		
Middle school	3874 (83.3)	4059 (80.2)		
College and above	643 (13.8)	928 (18.3)		
Occupation			116.338	0.000
Farmer	3426 (73.6)	3237 (64.0)		
Worker	241 (5.2)	271 (5.4)		
Staff in administrative institutions	101 (2.2)	154 (3.0)		
Business/company staff and else	884 (19.0)	1396 (27.6)		
Parity			109.220	0.000
0	2033 (45.2)	2690 (55.9)		
1	2344 (52.1)	2041 (42.4)		
≥2	122 (2.7)	83 (1.7)		
History of abnormal reproduction			3.038	0.081
No	2973 (63.9)	3318 (65.6)		
Yes	1679 (36.1)	1740 (34.4)		
Pre-pregnancy BMI			14.188	0.001
<18.5	857 (18.4)	898 (17.8)		
18.5–24.9	3338 (71.8)	3765(74.4)		
≥25.0	457 (9.8)	395 (7.8)		
Pregnancy comorbidity				
No	4386(94.3)	4783(94.6)	0.364	0.546
Yes	266(5.7)	275(5.4)		

BMI stands for body mass index.

### Adverse pregnancy outcomes of the two groups

The prevalence rates of preterm birth, low birth weight, macrosomia, SGA and LGA were 2.9%, 2.3%, 6.2%, 7.9% and 7.5%, respectively among the 5058 cosmetics users, and were 3.1%, 2.1%, 6.6%, 6.5% and 8.3%, respectively among the cosmetics non-users (Table 2). After adjustment for maternal age, residence, ethnicity, education level, occupation, parity, abnormal reproductive history, pre-pregnancy BMI and pregnancy comorbidity, a significant association of cosmetics use was found with SGA (aOR = 1.23, 95%CI 1.04 to 1.44), but not with the risk of preterm birth (aOR = 0.92, 95%CI 0.73 to 1.18), low birth weight (aOR = 1.17, 95%CI 0.88 to 1.55), macrosomia (aOR = 0.96, 95%CI 0.81 to 1.14) or LGA (aOR = 0.94, 95%CI 0.80 to 1.09).

**Table 2:** Adverse pregnancy outcomes for the cosmetics non-users and users.

Pregnancy outcomes	Cosmetics non-users (n = 4652)	Cosmetics users (n = 5058)	OR (95%CI)	
			Unadjusted	Adjusted^a^
Preterm birth^b^				
Yes	146(3.1)	148(2.9)	0.93(0.74–1.17)	0.92(0.73–1.18)
No	4506(96.9)	4910(97.1)		
Birth weight				
Low birth weight^c^	96(2.1)	118(2.3)	1.12(0.86–1.48)	1.17(0.88–1.55)
Normal birth weight	4247(91.3)	4626(91.5)		
Macrosomia^c^	309(6.6)	314(6.2)	0.93(0.79–1.10)	0.96(0.81–1.14)
Birth weight and gestational age				
SGA^c^	301(6.5)	399(7.9)	1.23(1.05–1.43)*	1.23(1.04–1.44*)
AGA	3965(85.2)	4282(84.7)		
LGA^c^	386(8.3)	377(7.5)	0.90(0.78–1.05)	0.94(0.80–1.09)

Results are presented as odds ratios (OR) with 95% confidence intervals (95%CIs). SGA stands for small for gestational age, AGA stands for appropriate for gestational age, LGA stands for large for gestational age. ^a^Analyses were adjusted for maternal age, residence, ethnicity, education level, occupation, parity, abnormal reproductive history, pre-pregnancy body mass index and pregnancy comorbidity. ^b^The odds ratio for preterm birth was calculated by binary logistic regression. ^c^The odds ratio for low birth weight/macrosomia and SGA/LGA was calculated by multinomial logistic regression.

### Dose-response relationships between frequency of cosmetics use and adverse pregnancy outcomes

According to categorization by frequency of cosmetics use, 350 (6.9%), 892 (17.6%) and 3816 (75.4%) of the 5058 pregnant women reported cosmetics use ≥5, 3–4 and 1–2 times per week, respectively (Table 3). Except for SGA, we found no significant differences in other adverse pregnancy outcomes between the cosmetics users and non-users in the dose-response analyses. The prevalence rate of SGA in cosmetics users at frequency of 1–2, 3–4, and ≥5 times per week were 7.7%, 7.6%, 10.3%, respectively. A positive dose-response relationship between frequency of cosmetics use and SGA was observed, although a borderline association was found with cosmetics use at low frequency (aOR = 1.18, 95%CI 0.99 to 1.40) or moderate frequency (aOR = 1.23, 95%CI 0.92 to 1.64). High frequency (≥5 times per week) of cosmetics use was related to a higher risk of SGA (aOR = 1.83, 95%CI 1.25 to 2.69).

**Table 3:** The dose-response relationships between frequency of cosmetics use and adverse pregnancy outcomes.

Pregnancy outcomes	Cosmetics non-users (n = 4652)	Cosmetics users (times/week)			OR (95%CI)^d^		OR (95%CI)^e^		OR (95%CI)^f^	
		1–2(n = 3816)	3–4(n = 892)	≥5(n = 350)	Unadjusted	Adjusted^a^	Unadjusted	Adjusted^a^	Unadjusted	Adjusted^a^
Preterm birth^b^										
Yes	146(3.1)	113(3.0)	26(2.9)	9(2.6)	0.94(0.73–1.21)	0.95(0.73–1.22)	0.93(0.61–1.42)	0.87(0.55–1.37)	0.82(0.41–1.61)	0.90(0.45–1.79)
No	4506(96.9)	3703(97.0)	866(97.1)	341(97.4)						
Birth weight										
Low birth weight^c^	96(2.1)	91(2.4)	17(1.9)	10(2.9)	1.16(0.86–1.55)	1.18(0.87–1.59)	0.91(0.54–1.54)	0.98(0.57–1.69)	1.39(0.72–2.70)	1.60(0.82–3.11)
Normal birth weight	4247(91.3)	3484(91.3)	824(92.4)	318(90.9)						
Macrosomia^c^	309(6.6)	241(6.3)	51(5.7)	22(6.3)	0.95(0.80–1.13)	0.97(0.81–1.16)	0.85(0.63–1.16)	0.92(0.67–1.25)	0.95(0.61–1.49)	1.00(0.62–1.60)
Birth weight and gestational age										
SGA^c^	301(6.5)	295(7.7)	68(7.6)	36(10.3)	1.20(1.01–1.42)*	1.18(0.99–1.40)	1.19(0.90–1.57)	1.23(0.92–1.64)	1.68(1.16–2.41)*	1.83(1.25–2.69)*
AGA	3965(85.2)	3246(85.1)	753(84.4)	283(80.9)						
LGA^c^	386(8.3)	275(7.2)	275(8.0)	31(8.9)	0.87(0.74–1.02)	0.90(0.76–1.06)	0.97(0.74–1.26)	1.00(0.76–1.32)	1.13(0.77–1.65)	1.25(0.84–1.87)

Results are presented as odds ratios (OR) with 95% confidence intervals (95%CIs). SGA stands for small for gestational age, AGA stands for appropriate for gestational age, LGA stands for large for gestational age. ^a^Analyses were adjusted for maternal age, residence, ethnicity, education level, occupation, parity, abnormal reproductive history, pre-pregnancy body mass index and pregnancy comorbidity. ^b^The odds ratio for preterm birth was calculated by binary logistic regression. ^c^The odds ratio for low birth weight/macrosomia and SGA/LGA was calculated by multinomial logistic regression. ^d^Odds ratio of adverse pregnancy outcomes compared cosmetics users (1–2 times/week) to cosmetics non-users. ^e^Odds ratio of adverse pregnancy outcomes compared cosmetics users (3–4 times/week) to cosmetics non-users. ^f^Odds ratio of adverse pregnancy outcomes compared cosmetics users (≥5 times/week) to cosmetics non-users.

## Discussion

This prospective cohort study showed maternal personal use of cosmetic products during pregnancy did not raise the risk of preterm birth, low birth weight, macrosomia, or LGA. However, a 23% increased risk of SGA was observed in cosmetic users. Moreover, a positive dose-response relationship was found between frequency of cosmetics use and SGA by all pregnant women in the sample, although a borderline association was found with cosmetics use at low or moderate frequency. This relationship was particularly evident in pregnant women with high frequency of cosmetics use. After adjustment of potential confounders, cosmetics users with low frequency, moderate frequency and high frequency were at an 18%, 23% and 83% increased risk for having SGA infants compared with cosmetics non-user, respectively. As we know, we were the first to examine the correlation between maternal personal exposure to cosmetic products during pregnancy and the risks of adverse pregnancy outcomes. This was a large-size longitudinal study based on the entire pregnant woman population in Zhuzhou and Xiangtan Cities, from whom the cosmetics users and non-users were selected. Moreover, the follow-up rate was very high, as only 45 pregnant women (0.5%) were lost. Thus, this study was not largely affected by selection bias or follow-up bias. In addition, the large sample size, reliable data collection and strong demonstrability for causality can accurately reflect the influence of maternal cosmetics use during pregnancy on the adverse pregnancy outcomes.

However, our results on preterm birth and low birth weight are inconsistent with previous literatures focused on occupational exposure^15,17^. A report from New York State shows maternal occupational exposure to cosmetic products is correlated with a higher risk of low birth weight^15^. A meta-analysis conducted by Kim concludes that cosmetologists are under higher risk of preterm birth than the general population^17^. The inconsistency in these studies may be attributed to the differences in cosmetics diversity, intensity and duration between personal and occupational exposures. Our findings on SGA are consistent with recent studies that maternal occupational exposure to cosmetic products during pregnancy will increase the risk of SGA^14,16^. A retrospective population-based study suggests the risk of SGA among cosmetologists’ offspring was raised by 40% compared to other working women^16^. Though we focused on the personal exposure to cosmetic products, our findings were similar to the research on occupational exposure. However, to date, there is no study exploring the relationship between macrosomia or LGA and cosmetic products exposure.

In our study, high-frequency use of maternal cosmetics was associated with SGA, but not with preterm birth or low birth weight. The reason may be that SGA is a general consideration of the effects of gestational age and sex on birth weight, and is more sensitive than preterm birth and birth weight and is likely to show statistical differences between cosmetics users and non-users. The relationship between cosmetics exposure and SGA is biologically plausible. The physiologic mechanism may be related to the known harmful effects of hazardous chemical substances in cosmetic products (e.g., phthalates, organic solvents, heavy metals) during fetus development. Two population-based birth cohort studies confirm that maternal exposure to cadmium, mercury, and arsenic during pregnancy would increase the risk of SGA^21,22^. Toxic metals can freely pass the placental barrier, transferring from the mother to the embryo, and accumulate in fetal tissues. These metals can alter the placental blood flow and endocrine function and injure the placental tissues, thereby hindering the nutrient transport to the fetus^23^. Since the fetal period is very critical for growth and development, the effect of any toxic substances including heavy metals is the most sensitive in this period and will lead to intrauterine growth retardation^24–26^.

Moreover, the prevalence rates of preterm birth and low birth weight are slightly lower than reported by a recent *World Health Organization Global Survey on Maternal and Perinatal Health* (4.8% and 3.6%, respectively)^27^. A recent large-scale study involving 28164 women in Shanxi Province from China shows the prevalence rate of low birth weight has decreased from 4.1% in 2010 to 2.6% in 2013^28^. Therefore, the prevalence rates of preterm birth and low birth weight in our study are similar to these studies. The prevalence rate of SGA in our study is close to the European level (4.6–15.3%)^29^, and the prevalence rates of LGA and macrosomia are close to the levels in developed countries^30^.

The present study presents no association between personal cosmetics use and preterm birth, low birth weight, macrosomia, or LGA, but shows that high-frequency cosmetics use is related to an increased risk of SGA. Nevertheless, maternal occupational exposure to cosmetic products would increase the risk of other adverse pregnancy outcomes, such as perinatal death, miscarriage, preterm birth, and low birth weight^13,15,17^. Therefore, cosmetics use during pregnancy needs to be cautious. Antenatal care providers should provide women of childbearing age, especially pregnant women or women planning pregnancy, with relevant health and safety education to minimize personal exposure to hazardous chemical substances in cosmetic products. Women should reduce the frequency of cosmetics use as much as possible during pregnancy.

This study has several limitations. First, the observational nature of the study design made it subject to some residual confounding. Our estimates were adjusted for several variables, which, however, excluded maternal stature, weight gain during pregnancy, nutritional intake, folic acid supplementation, or exposure to environmental pollutants, since information on these factors was unavailable in our study. These factors are reportedly related to an increased risk of low birth weight, SGA, or preterm birth^31–36^. Second, due to the difficulty in obtaining exact information about exposure variables, the frequency of cosmetics use throughout the pregnancy was evaluated according to self-reported general use habits of the pregnant women. During the cosmetics exposure measurement, we only focused on the average times of cosmetics use per week, but not the types or amount of cosmetic products per use. The information acquisition approach was simple and convenient, but self-reported exposure measurement inevitably had some inherent limitations. On one hand, the measurement accuracy of cosmetics exposure was decided by the subjective cooperativeness of the participants, which was not as objective as instrumental measurement; on the other hand, the recall of past cosmetics exposure might result in recall bias in the exposure data. Such measurement bias might affect the risk assessment of relationship between cosmetics use and adverse pregnancy outcomes to some extent. In addition, since the type of cosmetic products was not analyzed, we did not clarify which cosmetic products increased the risks of SGA, which should be clarified by more research and more human data. Third, the cosmetics ingredients and concentrations both differ among countries because of the differences in cosmetics hygienic criterias and provisions. Usage patterns of cosmetic products (e.g., frequency of use, types per use, amount per use) also significantly differ among people in different countries. Hence, the generalizability of our findings may be limited by the homogeneity of this cohort, which contained only Chinese women.

In conclusion, personal cosmetics use is not related to preterm birth, low birth weight, macrosomia, or LGA, but high-frequency cosmetics use will increase the risk of SGA. Further research is required to determine which cosmetic products may account for the higher risk of SGA.

## Data Availability

The datasets generated during and/or analysed during the current study are not publicly available duo to the personal privacy of subjects but are available from the corresponding author on reasonable request.
